# Speech and Language Practitioners’ Experiences of Commercially Available Voice-Assisted Technology: Web-Based Survey Study

**DOI:** 10.2196/29249

**Published:** 2022-01-05

**Authors:** Pranav Kulkarni, Orla Duffy, Jonathan Synnott, W George Kernohan, Roisin McNaney

**Affiliations:** 1 Action Lab Department of Human Centred Computing Monash University Clayton Australia; 2 School of Health Sciences Ulster University Newtonabbey United Kingdom; 3 School of Computing Sciences Ulster University Newtonabbey United Kingdom; 4 Institute of Nursing and Health Research Ulster University Newtonabbey United Kingdom

**Keywords:** speech and language therapy, voice-assisted technology, professional practice, rehabilitation, speech therapy, health technology, mobile phone

## Abstract

**Background:**

Speech and language therapy involves the identification, assessment, and treatment of children and adults who have difficulties with communication, eating, drinking, and swallowing. Globally, pressing needs outstrip the availability of qualified practitioners who, of necessity, focus on individuals with advanced needs. The potential of voice-assisted technology (VAT) to assist people with speech impairments is an emerging area of research but empirical work exploring its professional adoption is limited.

**Objective:**

This study aims to explore the professional experiences of speech and language therapists (SaLTs) using VAT with their clients to identify the potential applications and barriers to VAT adoption and thereby inform future directions of research.

**Methods:**

A 23-question survey was distributed to the SaLTs from the United Kingdom using a web-based platform, eliciting both checkbox and free-text responses, to questions on perceptions and any use experiences of VAT. Data were analyzed descriptively with content analysis of free text, providing context to their specific experiences of using VAT in practice, including barriers and opportunities for future use.

**Results:**

A total of 230 UK-based professionals fully completed the survey; most were technologically competent and were aware of commercial VATs (such as *Alexa* and *Google Assistant*). However, only 49 (21.3%) SaLTs had used VAT with their clients and described 57 use cases. They reported using VAT with 10 different client groups, such as people with dysarthria and users of augmentative and alternative communication technologies. Of these, almost half (28/57, 49%) used the technology to assist their clients with day-to-day tasks, such as web browsing, setting up reminders, sending messages, and playing music. Many respondents (21/57, 37%) also reported using the technology to improve client speech, to facilitate speech practice at home, and to enhance articulation and volume. Most reported a positive impact of VAT use, stating improved independence (22/57, 39%), accessibility (6/57, 10%), and confidence (5/57, 8%). Some respondents reported increased client communication (5/57, 9%) and sociability (3/57, 5%). Reasons given for not using VAT in practice included lack of opportunity (131/181, 72.4%) and training (63/181, 34.8%). Most respondents (154/181, 85.1%) indicated that they would like to try VAT in the future, stating that it could have a positive impact on their clients’ speech, independence, and confidence.

**Conclusions:**

VAT is used by some UK-based SaLTs to enable communication tasks at home with their clients. However, its wider adoption may be limited by a lack of professional opportunity. Looking forward, additional benefits are promised, as the data show a level of engagement, empowerment, and the possibility of achieving therapeutic outcomes in communication impairment. The disparate responses suggest that this area is ripe for the development of evidence-based clinical practice, starting with a clear definition, outcome measurement, and professional standardization.

## Introduction

### Background

Speech and language therapy (SLT) is an allied health profession concerned with the assessment, diagnosis, and treatment of a range of both communication and swallowing disorders [1]. Speech and language therapists (SaLTs) support a broad range of people within pediatric and adult services (eg, early language development, learning disabilities, Parkinson disease, stroke, and traumatic brain injury) and work within a wide range of settings (eg, schools, homes, care homes, hospitals, and prisons) [2]. SaLTs are responsible for delivering a range of evidence-based therapeutic interventions to support the clinical needs of their service users, related to improving communicative ability and managing eating, drinking, and swallowing difficulties. The ultimate goal of any therapeutic program is the generalization of principles learned in the clinical context to a person’s everyday life [3]. Despite providing a core service within rehabilitative and long-term care—particularly in acquired or degenerative neurological conditions—SLT, similar to many other services, has been affected by funding cuts. A survey by the Royal College of Speech and Language Therapists (RCSLT) suggests that over 80% of services in the National Health Service (NHS) face reduced staffing, narrowing scope of services and, in 8% of the services, abolishment of services altogether [4].

There is a potential for appropriate technology-based solutions to assist in reducing the burden on staff and widening access to care services. One of the key areas where technology has impacted SLT has been in the development of *augmentative and alternative communication* (AAC) devices. AAC is a term used to describe various methods of aided communication, including nonverbal strategies such as gestures or body language, the use of picture books or communication charts, or a range of different technologies that can act as a substitute vocal communication aid [5]. The types of technologies used for AAC are diverse with varying complexities—from equipment with simple text to speech functions, picture-based *buttons* that relay messages when pressed, to eye gaze technology for those who are physically unable to physically interact with a system. The development of AAC apps [6-8] that can be downloaded from commercial app platforms and installed on personal mobile devices has seen a recent rise in popularity, with the purpose of bringing the benefits of AAC to a wider range of individuals by reducing the associated costs.

Although AAC is a well-established field with clinically proven benefits, researchers have also been investigating other areas of technology innovation within the field of SLT, particularly those that exploit the benefits of low-cost, off-the-shelf consumer technology. For example, research has explored the role of technology in supporting SLT for people with aphasia—a communication difficulty affecting the expression or comprehension of spoken and written language [9-15]. One study explored an approach toward making paper-based resources, such as worksheets, stickers, and photographs more interactive by enabling therapists to customize content with personally meaningful and useful audio clips [11]. Another study developed a context-aware system that provided the user with relevant word lists to select from, depending on their location [15]. Similarly, Williams et al [13] explored the potential for providing in situ support for the access of vocabulary during conversation, using head-mounted wearable technologies, such as Google Glass, and wrist-mounted touchpads for easy navigation. In another study, Google Glass was used to provide volume training for people with Parkinson disease [16], providing real-time feedback on the users’ speech volume by indicating that a predefined target was achieved. The participants provided positive feedback and described the benefits of the voice interaction functionality for technology access. These studies highlight the potential of technology-assisted SLT to address client needs cost-effectively.

### Voice-Assisted Technology

Voice assistants are software applications (eg, Siri [17], Google Assistant [18], and Amazon Alexa [19]), which have become increasingly popular in smartphones, computers, tablets, and purpose-built speakers. They can interpret human speech and allow interaction with the technology through spoken commands, allowing users to complete a variety of tasks such as setting alarms, searching for information on the web, playing music, providing weather updates, and telling the time. These devices allow infinite attempts for the user to practice their speech and commands and will actively acknowledge if it has misunderstood the attempt, which can be a prompt to modify speech.

Recent figures show that almost 29% of the population in the United Kingdom [20] has access to a smart speaker. Another report suggests that the COVID-19 lockdown has increased interaction with voice assistants in the United Kingdom [21]. As such, the popularity of these devices is growing and they are being widely accepted. Similar smart speaker ownership has also been recorded in other countries, such as Australia [22] and the United States [23]. The older adult population (aged ≥60 years) accounts for approximately 20% of smart speaker ownership, with almost 60% of these consumers using the device every day [24]. The technology offers hands-free access and naturalistic voice interaction, a beneficial means of interacting with the device for those with physical disabilities or lower levels of technology literacy [25]. These features have motivated research in the health care sector, and recent years have seen an emergence in research that explores the use of voice-assisted technology (VAT) to support people within these demographics.

Several studies have explored how diverse populations such as older adults [26], people with visual and physical disabilities [27-30], and people with speech impairments [31-33] use and interact with VAT. One study exploring the experiences of older adults’ use of Alexa reported positive first interactions when using the device, owing to the simplicity of speech-based communication, but highlighted the need for better device training and the privacy, security, and financial concerns raised by the participants [26]. Studies exploring the experiences of people with disabilities [27,28] and health concerns [27] more broadly have also focused their attention on Alexa as a VAT of interest, with retrospective qualitative analyses being conducted on a significant number of consumer reviews of Alexa-enabled smart speakers on Amazon. These studies have demonstrated increased independence and empowerment when using the device. Although not the primary focus of their analysis, Pradhan et al [28] also explicitly described successful use in many cases of customers who had reported speech difficulties.

One recent study explicitly focused on VAT use among people with speech impairments. A survey conducted on 290 people with Parkinson disease (78% of whom were assessed to have mild to moderate speech impairment) explored their access and use of VAT, including whether they had noticed any changes to their speech because of using the device [32]. The authors found that as many as 25% of the participants reporting changes to their speech had noticed improvement, indicating a clear potential direction for future work exploring VAT as a tool to support outcomes relevant to SLT. Participants in this study, who were primarily in the range 65-74 years, also had high levels of success when using VATs (most used the device regularly and rarely had to repeat themselves), a finding further echoed by McNaney et al [29]. Further studies have investigated the success rates of different VATs (Cortana, Microsoft Inc; Alexa; and Siri) for people with dysarthria (a group of neurological speech disorders affecting intelligibility), finding recognition accuracy in the range of 50%-60% with single prerecorded samples [33]. They did not report on the severity or etiology of the speakers’ dysarthria; however, the study was not conducted with live speakers in naturalistic settings, which would be required to accurately draw conclusions about how successful dysarthric users may be in voice interface interactions. Another study explored how the application of adaptive voice recognition (ie, systems that learn the user’s voice over a series of sessions) could have promise for improving the accessibility of VAT for users with speech difficulties [31].

Therefore, although research into the space of VAT for people with impaired speech is emerging, most studies have focused on the end users’ perceptions and how the device is used *out of the box*. To the best of our knowledge, there has been no previous work exploring the perceptions of VAT by SaLTs and its use in clinical practice.

### Study Aims

The primary aim of this study is to gather a preliminary overview of how SaLTs and their clients have been using VAT. We wanted to understand the potential opportunities and challenges of VAT use from SaLTs’ perspectives and gather an understanding of current use cases within SLT practice, whether this be directed by their client or explicitly used by therapists for clinical reasons. We aim to answer the following research questions: (1) If SaLTs have used VAT with their clients, what was their experience of its use? (2) If VAT was not being used currently, what were the possible reasons for this? (3) What are the perceived benefits, risks, and barriers to using VAT in SLT?

By addressing these questions, we aim to provide a foundation for future research, which will explore how VAT might be used in clinical practice, the types of clients it might be useful for, and the types of activities that clinicians might perform with VAT to support therapeutic delivery.

## Methods

### Survey Design

We developed a survey to gather the experiences of the SaLTs from the United Kingdom about how they and their clients had been using VATs to support their clinical needs and to understand the possible barriers and opportunities toward the future use of VATs in clinical practice. A draft survey was pilot-tested with 3 academic staff members at Ulster University and 2 SaLTs, with minor amendments to improve the clarity and flow of the questions based on their feedback.

The finalized version consisted of 23 questions (please note that participants were not required to respond to every question) and consisted of three sections:

1. Demographics, such as age, job title, years of experience, and clinical caseloads.
2. Digital skills assessment: this was adapted from The Tech Partnership’s Basic Digital Skills framework (reuse permission was granted) [34]. The assessment provided statements describing 11 digital tasks spanning areas, including managing information, communicating, transacting, problem solving, and creating. For example, a *managing information* digital skill is *find a website I have visited before*. For each of the 11 statements, respondents were asked to indicate whether they could or could not complete the digital task described in the statement.
3. VAT familiarity, use, and the participants’ opinions on the potential barriers, benefits, and impacts of using VAT to support client’s needs in their clinical practice.

**Figure 1:**
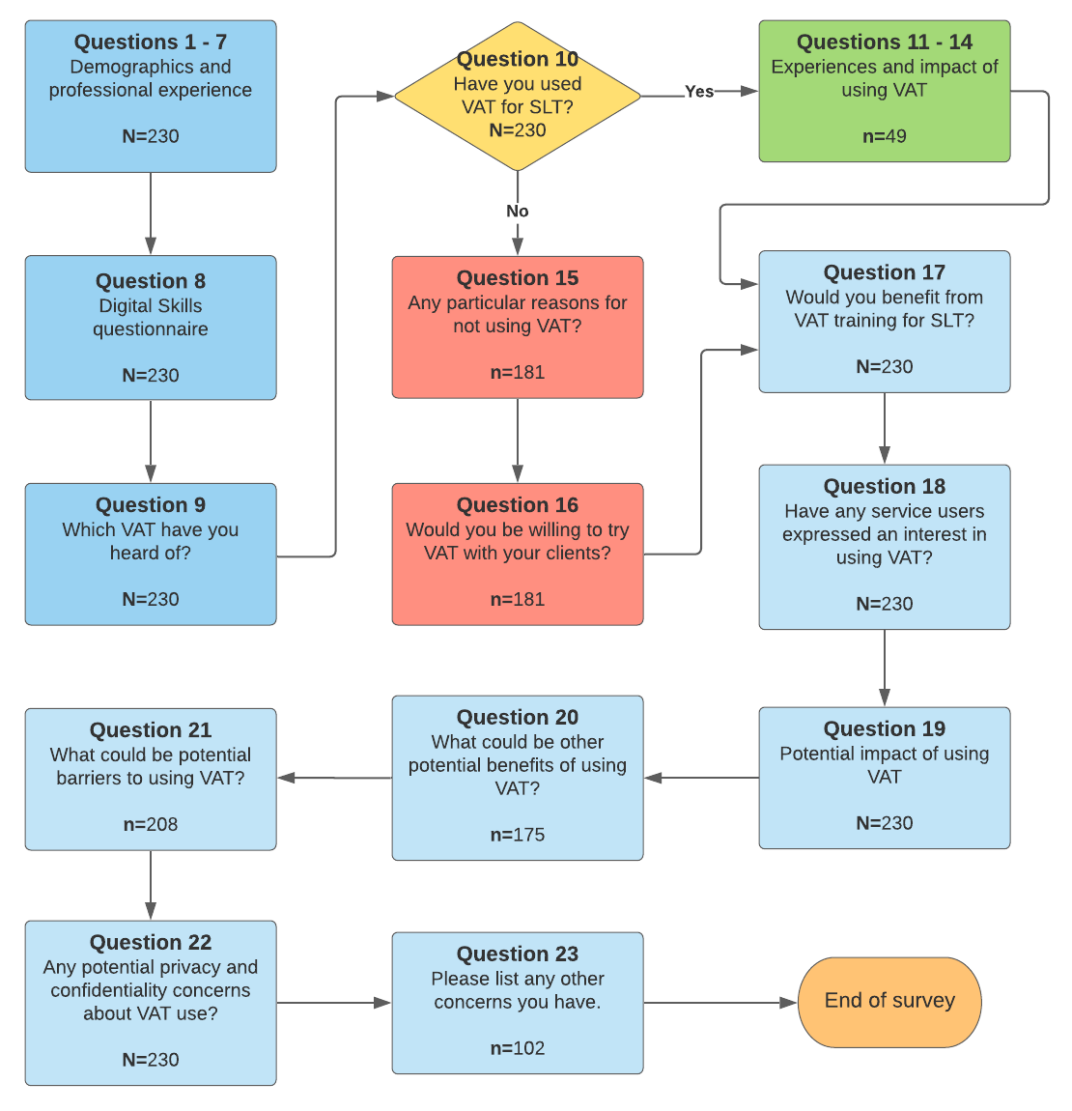
Survey flow diagram: starting at the top left, the diagram shows elements of the survey with skip logic to avoid irrelevant directions of questioning (the number of respondents to each element have been provided). SLT: speech and language therapy; VAT: voice-assisted technology.

The survey questions were designed to obtain both quantitative and qualitative insights from the participants using a mix of checkbox and free-text questions. It was developed and distributed using Qualtrics, a web-based survey tool [35]. Figure 1 shows a breakdown of the questions asked during the survey.

### Study Ethics, Population, and Recruitment

The study was peer-reviewed, and ethical approval was obtained from the Ulster University Institutional Research ethics committee. The web-based survey was distributed to members of the RCSLT by advertising on their social media platforms (Facebook and Twitter) in January 2019. Following the social media recruitment phase, 111 clinical excellence networks in RCSLT were contacted in February 2019, and the survey was disseminated through their membership using a snowball sampling technique. Details of the study were presented on the welcome page of the survey, including information about the purpose of the study, the length of time required to complete it, data storage, and anonymity. The participants were informed that consent was provided through the completion of the survey. The intended sample size was based on the number required to obtain 90% confidence with –5% to +5% margin of error in estimating proportions: the exact calculation was a sample size of 289.

### Data Analysis

As the survey consisted of both checkbox and free-text responses, the study used a mixed methods approach for analysis. For each checkbox question, the total number of participants responding to each possible option (ie, count) was collected and the percentage (of overall respondents to that question) was provided. Summative content analysis [36] was used for qualitative free-text responses [37]. The responses from each free-text question were collated and analyzed separately by 2 researchers to identify the themes. Any disagreements were resolved through discussion until a decision was made on the final set of themes. The themes were summarized, with several responses relating to each theme available for the analysis.

## Results

### Overview

The survey received responses from 261 respondents. Partially completed survey responses (31/261, 11.9%) were excluded, as ethically we only considered participants who completed the survey as fully consenting to the study. This left 230 fully completed surveys for the analysis. Most of the respondents (223/230, 96.9%) were women, with a large proportion (102/230, 44.3%) aged <35 years; 64 (27.8%) respondents were aged between 35 and 44 years, 41 (17.8%) respondents were aged between 45 and 54 years, and the remaining 23 (10%) respondents were aged >55 years.

### Professional Experience Demographics

Most of the respondents (227/230, 98.7%) were practicing SaLTs, with varying years of experience in the field. Very few respondents (3/230, 1.3%) worked in academia, in research, and teaching. They reported a mean work experience of 13 years (SD 9.45 years). The majority (184/230, 80%) worked for the UK NHS: half of this population (92/184, 50%) were early career practitioners working in NHS bands 5 and 6, whereas the other half (92/184, 50%) were more experienced practitioners working in NHS bands 7 and above. The rest (46/230, 20%) did not work for the NHS. The respondents worked across a wide range of clinical caseloads (Table 1).

Some participants (27/230, 11.7%) reported *other* caseloads in the form of a free-text response, including social, emotional, and mental health (8/230, 3.5%); head and neck cancer (8/230, 3.5%); early language development (7/230, 3%); selective mutism (2/230, 0.9%); and general research (2/230, 0.9%).

**Table 1:** Reported clinical caseloads (N=230).

Caseload	Count, n (%)
Dysphagia (swallowing difficulty)	103 (44.8)
Augmentative and alternative communication	100 (43.5)
Acquired communication disorders	90 (39.1)
Learning disabilities	90 (39.1)
Autistic spectrum disorder	87 (37.8)
Progressive neurological conditions	70 (30.4)
Developmental language disorders	69 (30)
Speech sound disorders	67 (29.1)
Dementia	57 (24.8)
Dysfluency	47 (20.4)
Voice	34 (14.8)
Deafness	24 (10.4)
Cleft lip and palate	18 (7.8)
Others	27 (11.7)

### Digital Skills

An adapted version of the digital skills questionnaire [34] was included in the survey. This consisted of 11 statements describing a range of digital skills. These skills ranged from using a search engine to look for information on the web to creating something new from existing web-based images, music, or videos. For each statement, respondents were asked to indicate whether they could or could not perform the technology-related activity the statement referred to, with the purpose of gauging the digital competence of the respondents. More than 95.7% (220/230) of the participants possessed 9 out of the 11 skills. The highest rated skills (229/230, 99.6%) were the following: *using a search engine to look for information online, download a photo you found online, find a website you have visited before, send a personal message to another person via email or online messaging service, buy items or services from a website, and complete online application forms which include personal details*. The skill *create something new from existing online images, music, or video* had the lowest number of participants indicating they could complete it (179/230, 77.8%).

### VAT Awareness and Use

Alexa was the most common VAT that the participants had heard of (229/230, 99.6%), followed closely by Siri (226/230, 98.3%) and Google Assistant (201/230, 87.4%). Most of the participants (181/230, 78.6%) had not used VAT with their SLT clients but 92.7% (166/181) of these indicated that they would like to use it in the future. Most participants (198/230, 86.1%) also indicated that they would benefit from training using VAT with their SLT clients. Of these 198 participants, 167 (84.3%) participants provided additional information about their training needs, with many participants (97/167, 58.1%) discussing a need for general information and awareness training, some participants (25/167, 15%) wanting structured information about using VAT with specific user groups and specific activities that could be conducted with the VAT, and few participants (9/167, 5.4%) interested in learning about how the technology is being used in the SLT community through real-world examples and in understanding the technical aspects of VATs and how they could improve the intelligibility of speech (6/167, 3.6%).

Participants who responded that they had never used VAT with their SLT clients were asked to provide the possible reasons for this (Table 2).

**Table 2:** Reasons for not using voice-assisted technology (N=181).

Reason	Count, n (%)
I have never had the opportunity to use it	131 (72.4)
I have not had any training	63 (34.8)
I do not know what technology is available	62 (34.2)
Technology is too expensive	32 (17.7)
I do not think there would be any benefit from speech and language therapy	20 (11)
Technology is too complicated	16 (8.8)
I am not interested in using technology	5 (2.8)
Other (please specify)	31 (17.1)

Other reasons were provided as free-text responses. These were mainly centered on barriers to accessing these types of technologies within the current digital infrastructure of the work environment (23/181, 12.7%), for example, “NHS IT puts up too many barriers to using with patients,” “Poor Wi-Fi in NHS premises,” and “lack of availability of up-to-date technology in my workplace.” Other reasons identified were reluctance to use technology by their clients (6/181, 3.3%) and privacy concerns (2/181, 1.1%). A small number of participants indicated that they had not used and would never want to use VAT with their SLT clients in the future (15/230, 6.5%).

### Barriers to Using VAT

The entire cohort of the survey participants (N=230) was invited to provide free-text qualitative responses around what they perceived might be the potential barriers to using VAT in therapy. A total of 208 (90.4%) respondents provided further information.

Over half of these respondents (105/208, 50.5%) had concerns about the devices’ ability to understand SLT clients’ speech, which they felt could be demotivating: “If a client's speech is too unclear they may receive negative feedback which would be disheartening and may lead to low self-esteem” and “Accents, speech sound errors not recognised by a computer but would be recognisable to humans. Can negatively impact confidence.” Very few respondents (3/208, 1.4%) also stated that device use could reinforce incorrect speech or pronunciation, which was a genuine concern. One participant stated, “Sometimes there are subtleties to speech which we are working on *e.g.* lateralization. They could be understood by Alexa/Siri which may reinforce incorrect productions.”

Some of the respondents (56/208, 26.9%) mentioned the lack of technical skills and/or the ability to use the devices by them and their clients as barriers. They provided examples such as “My lack of technological knowledge, time in what is a very time-intensive area in terms of assessing, researching, liaison, implementation and monitoring” and “Inherent difficulties with technology - things not being plugged in correctly, set up correctly or carers not having sufficient skill to rectify any issues as they arise.” Similarly, psychological barriers were also identified by a few respondents (29/208, 13.9%). Older clients were considered potentially unwilling to use the technology because of unfamiliarity or being *scared* of technology use: “Older patients tend to be more resistant to using/learning new technology” and “Tech knowledge and confidence of service users and/or their supporters. Resistance to the concept of tech in some clients.” A few respondents (8/208, 3.8%) mentioned that learning trigger words could be challenging, stating:

The models available at the moment require very specific multi-word trigger phrases which lots of people with LD (learning disability) wouldn't be able to get right every time. Could lead to frustration if you need to repeat the phrase because it wasn't picked up originally.

Another barrier that many respondents (73/208, 35.1%) identified was the cost and availability of the devices for therapy, providing examples such as “Expensive for NHS and patients to purchase” and “Access - often elderly patients may not have suitable equipment/devices.” A few respondents (30/208, 14.4%) also mentioned that setting up the infrastructure to support the devices could be challenging. Information technology support, internet, troubleshooting, and maintenance were some of the infrastructural challenges mentioned: “IT systems in Local Authorities and *NHS*. Poor internet speeds in rural areas” and “Resources, information governance, need to be appropriately used and managed.” Other respondents (11/208, 5.3%) had privacy and security concerns, which were seen to be a potential barrier to VAT use: “Some people refuse Alexa because they don’t want to feel ‘big brother’ is watching them all the time” and “believing that the device owner (*E.g.* Google/Amazon) are collecting and saving your data.”

### Privacy and Security Concerns

We further explored possible privacy, security, and confidentiality concerns with all participants by asking them to rate their level of concern: not concerned (75/230, 32.6%), slightly concerned (132/230, 57.4%), or very concerned (23/230, 10%). Participants were then asked to provide free-text responses highlighting any concerns they might have (102/230, 44.3% provided additional information).

Many respondents (38/102, 37.2%) discussed the *always on* nature of VAT and the fact that these devices are *always listening*:

I would be concerned that the microphone seems to be always listening to all conversations, therefore impacting on privacy.

There are still reports of voice assistants being used as eavesdroppers, compiling information on users etc. I personally wouldn't have one in the house but can see use for people if they are happy to take that risk.

Other respondents (21/102, 20.6%) were concerned about who had access to the data, especially considering these are commercial devices with data being collected and stored by large-scale technology companies: “It is unclear how that data is used, who has access to it and/or can purchase it*.*” Some respondents (12/102, 11.8%) were also concerned about data use, stating, “Use of voice data is not clearly articulated by companies such as Amazon and Google.” This was further elaborated, with 8.8% (9/102) of the respondents stating their concern over targeted advertisements: “They're always listening and then provide tailored adverts.” In a similar sense, several respondents (21/102, 20.6%) were concerned about data storage and security: “VAT can make anyone vulnerable to leaks of personal information so an added difficulty in communicating could increase that person's vulnerability if not managed appropriately.”

Regulatory and General Data Protection Regulation (GDPR) issues were also discussed (8/102, 7.8%):

I am concerned that organisations will put hurdles in the way which would prevent people benefiting from voice assisted technology. For example, being told that a device can’t be purchased or used due to GDPR etc.

Finally, some respondents (5/102, 4.9%) expressed their concerns over unsupervised access and accidental purchases using the devices: “children accessing internet with devices potentially unsupervised” and “buying products or apps without awareness*.*”

### Experiences of Using VAT in Clinical Practice

#### Overview

A total of 49 respondents reported that they had used VAT with their clients. These respondents were asked a set of free-text questions to gather details about their experiences. They were explicitly asked the following three questions:

1. Please provide some detail about your experience of using VAT for SLT. Please describe the types of service user you have used VAT with.
2. Please provide some detail about how you used VAT with service users.
3. What was the impact of using VAT with service users?

A total of 57 cases were discussed by the respondents, as some reported multiple use cases. There were 10 major client groups across adult and pediatric services that were discussed (Tables 3 and 4). Almost half of the respondents (28/57, 49%) had used the technology to support day-to-day tasks, such as setting reminders, playing music, and sending emails and text messages. Many respondents (21/57, 37%) reported using VAT specifically for SLT practice (ie, using it to support the training of explicit SLT strategies with their clients). Others reported using the devices for speech to text functionality (9/57, 16%), environment control (9/57, 16%), and to set up an AAC device (2/57, 3%). A handful of respondents also reported using it as a motivational tool for therapy (3/57, 5%), a tool for routine formation (1/57, 2%), and a tool for translation (1/57, 2%).

In terms of the impact of VAT use, the respondents reported a multitude of positive impacts. They reported increased client independence (22/57, 39%), accessibility (6/57, 10%), and confidence (5/57, 9%). Some respondents discussed the impact on their clients’ speech. They mentioned that their clients received feedback on their speech (9/57, 16%) and reported increased client communication (5/57, 9%) and sociability (3/57, 5%). A full breakdown of the findings is presented in Tables 3 and 4. We then contextualized and drew out the respondents’ experiences further through a narrative description of the data.

**Table 3:** Voice-assisted technology use cases.

Client group example and main use cases			Respondents, n (%)
**Dysarthria (n=18)**			
	SLT^a^ practice	10 (55)	
	Day-to-day tasks	5 (28)	
	Environment control	5 (28)	
	Speech to text	2 (11)	
**Augmentative and alternative communication** **(n=15)**			
	Day-to-day tasks	10 (67)	
	Environment control	4 (27)	
	Augmentative and alternative communication setup	2 (15)	
	Motivation tool	1 (7)	
**Aphasia (n=7)**			
	Speech to text	4 (57)	
	SLT practice	3 (43)	
	Day-to-day tasks	2 (28)	
**Learning disability (n=5)**			
	Day-to-day tasks	4 (80)	
	SLT practice	3 (60)	
**Mainstream school setting (n=3)**			
	Day-to-day tasks	3 (100)	
	Motivation tool	2 (67)	
	Speech to text	1 (33)	
	SLT practice	1 (33)	
**Traumatic brain injury (n=3)**			
	Day-to-day tasks	3 (100)	
	SLT practice	1 (33)	
**Apraxia (n=2)**			
	SLT practice	2 (100)	
	Speech to text	2 (100)	
**Cognitive communication disorder (n=2)**			
	SLT practice	1 (50)	
	Routine formation	1 (50)	
**Dementia (n=1)**			
	Day-to-day tasks	1 (100)	
**English as a second language (n=1)**			
	Translation tool	1 (100)	

^a^SLT: speech and language therapy.

**Table 4:** Impact of using voice-assisted technology.

Client group example and reported impacts		Respondents, n (%)
**Dysarthria (n=18)**		
	Increased independence	9 (50)
	Feedback on speech	6 (33)
	Increased engagement as technology is an everyday device	3 (17)
	Increased speed of task	2 (11)
	Increased quality of life	1 (5)
	Increased accessibility	1 (5)
**Augmentative and alternative communication** **(n=15)**		
	Increased engagement as technology is an everyday device	7 (47)
	Increased independence	5 (33)
	Increased accessibility	3 (20)
	Increased quality of life	2 (13)
	Increased communication	2 (13)
	Increased sociability	1 (8)
**Aphasia (n=7)**		
	Increased independence	3 (43)
	Feedback on speech	2 (29)
	Functional writing	1 (14)
	Increased sociability	1 (14)
	Increased confidence	1 (14)
**Learning disability (n=5)**		
	Improved communication	3 (60)
	Increased confidence	2 (40)
	Increased independence	1 (20)
	Increased engagement	1 (20)
**Mainstream school setting (n=3)**		
	Increased engagement as technology is an everyday device	2 (67)
	Increased confidence	1 (33)
**Traumatic brain injury (n=3)**		
	Increased independence	2 (67)
	Increased accessibility	2 (67)
	Increased confidence	1 (33)
**Apraxia (n=2)**		
	Increased independence	1 (50)
	Increased sociability	1 (50)
	Feedback on speech	1 (50)
**Cognitive communication disorder (n=2)**		
	Increased independence	1 (50)
	Increased engagement as technology is an everyday device	1 (50)
	Increased independence	1 (50)
**Dementia (n=1)**		
	Increased independence and sociability	1 (100)
**English as a second language (n=1)**		
	Improved communication with English as a second language student and time saving (as no need for a translator)	1 (100)

#### Dysarthria

Of the described use cases, dysarthria was the most common, with 18 therapists reporting using a range of VATs with their dysarthric clients. Dysarthria is a motor speech impairment caused by weakening or paralysis of the muscles used to produce speech. It often presents as slow or slurred speech, which can be difficult to understand. The therapists primarily discussed using VATs to support speech practice as a way to provide biofeedback to the client on their speech clarity. For example, 1 therapist described how a client “uses it to practice speaking with strategies to make her speech clearer because if Alexa understands her, she knows she is doing well”; another’s client “uses it to monitor volume and intelligibility and finds it objectively helpful.” One therapist discussed how their client had actively “identified the goal of being understood by Siri” as an outcome measure for their therapy. This process of enhancing the clients’ practice of speech and the ability to give clients continuous feedback on their speech was seen as particularly beneficial for this user group.

Several therapists also explicitly described working with dysarthric patients with specific neurological conditions such as Parkinson disease (“speech therapy to improve accuracy of speech to text recognition software”), multiple sclerosis (“used an Alexa which was used as a switch device to control items in his environment e.g. curtains, fan...increased independence and reduced frustration”), and motor neuron disease (“sending text messages, memos, calendar, email, web search...it kept the patients using their phones and felt less medical- minimal training required”). The day-to-day functional outputs provided by VAT devices (eg, using speech to text to write memos, searching for web-based information, and writing shopping lists) and the ability to support those with comorbid physical impairments (eg, because of a neurological condition or paralysis post stroke) by enhancing their ability to control the environment were frequently outlined by the therapists as improving the independence of individuals within this client group.

#### Augmentative and Alternative Communication

The second most discussed use cases that were provided centered on AAC users. A total of 15 therapists described using VATs in varying degrees with this client group. AAC refers to any communication method used to supplement or replace spoken or written speech production; however, in our case, therapists explicitly discussed digital AAC devices. Most use cases discussed how the therapists had helped their clients set up their voice output devices to provide commands, primarily to Alexa, thereby allowing their clients to access the functionalities of VATs through a computerized voice. This enabled their clients to complete everyday tasks, such as searching for information on the web or listening to music as well as to control their environments. For example, 1 therapist described it as follows:

enabled voice output devices to liaise with voice assisted technology to enable control and information gathering and sharing, e.g. with Nest to control heating; used with shopping apps like Amazon; request information about news, local events; to access leisure activities like music, television, Netflix...many many ways.

The therapist described how using the technology in this way “facilitated great independence [and] facilitated and maintained social contacts.” Several therapists (n=3) also discussed nonverbal clients who were users of AAC devices controlled through eye gaze. One therapist described setting up *pages* on a client’s device (ie, a visual page of icons or images on the device that the user can activate using eye gaze and blinking):

so that they can use Alexa to play music, or activate a disco ball (favourite toy)...enabled a little girl with progressive muscle weakness to control music and toys after losing hand function. Did this using mainstream technology [Alexa] that her parents were confident with and found acceptable and exciting to use.

Therapists describing use cases in this theme were very positive about the impacts of VAT on their clients’ lives, describing how it had “greatly improved their quality of life in all situations” and had led to improvements in the clients’ independence and confidence. There were also many comments stating that clients were motivated to engage with the technology as it was an everyday device: “users love it as it’s not a ‘disability device’- it is something everybody is using.”

#### Aphasia

Seven of the therapists described using VAT with clients with aphasia (a disorder affecting the ability to produce and comprehend spoken and written language). Primarily, it was used for its speech to text functionality to support written communication. One therapist described it as follows:

We used voice assisted tech for Google searching, writing emails and texts and writing stories...many of the reported increased confidence and self-esteem...Some of the people in the clinic even managed to get back into work as a result.

However, several therapists also described using it as a tool to facilitate spoken language tasks. For example, 1 therapist used it to “practice spoken language in a ‘real life’ setting and also [to support] comprehension (e.g. playing games with Alexa skills where they listen to instructions and then give verbal commands)” and another described its use at helping clients “generate clear and accurate sentences, format questions without hesitancy and learn how to phrase to get the best results.”

#### Learning Disability

Therapists working with people with learning disabilities (n=5) mainly discussed using VAT as a tool to support day-to-day tasks, such as playing music or searching for things on the web: “Alexa or Siri to enable music to be played or to search the web, used mainly with individuals with mild to moderate learning disabilities,” which was seen to be “very positive, provides control/independence, boosts confidence and gives further topic conversation with staff/family [as well as] increasing awareness of [their] own speech and how this is interpreted by others/Alexa.” Therapists also discussed using the device to specifically target language tasks such as phrase construction, supporting joint attention with a carer, and training conceptual understanding of cause and effect. One therapist described a specific activity that they had designed to use with Google Assistant: “service user makes a request ‘moo’, carer says ‘OK Google, play the sound of a cow’. Other farm animals and vehicles were also used.”

#### Mainstream School Setting

Similarly, within the mainstream school setting (ie, where therapists might have worked with children who had milder speech and language impairments or delays), we had 3 therapists using VAT primarily to keep the children engaged “as a motivation tool...students were more engaged as the technology is more relevant to their lives.” One therapist described using the technology creatively to “ask Alexa to make silly noises or to tell us a joke during classroom-based sessions” but it was mainly described as being used to search for things on the web or to play music:

It’s been incredible. The instant gratification has meant the children keep going back to use it. We had to get the school to buy an amazon music subscription.

#### Traumatic Brain Injury

Three therapists had used VAT with clients with traumatic or acquired brain injury, which is caused by sudden trauma in the brain (eg, from a sports injury or car crash). This type of injury can cause a range of issues that might be supported by an SLT, relating to an individual’s speech, language, writing, social communication, behavior, attention, planning, learning, and swallowing. All of the therapists were using VAT to support day-to-day activities, such as *turning on the radio, making phone calls, checking the weather*, *setting reminders, and calling* and reported improvements to confidence, independence, and access to technology. One therapist also used it to support *verbal reasoning/problem solving* by asking the client to find out and respond to information by using the device.

#### Apraxia

Similar to dysarthria, apraxia of speech can cause issues with speech intelligibility. Although both are motor speech disorders, apraxia is more concerned with the planning, sequencing, and coordination of speech production. Two therapists had used VAT (Alexa and Siri) to support speech therapy practice, in particular, “to provide biofeedback on how intelligible the clients’ speech is.” One therapist described it as follows: “Direct work on improving apraxia of speech, word production accuracy and improving impairment.” The technology’s ability to respond correctly to verbal commands was seen as a valuable way to *enable clients to practice clear speech at home with measurable outcomes.* Both therapists also used speech to text functions to enable their clients to create written notes, messages, and emails.

#### Cognitive Communication Disorder

There were 2 respondents who discussed use cases with clients experiencing cognitive communication disorders, which can cause difficulties in remembering information, staying on topic, and maintaining attention. One respondent used interaction with Alexa to support *generating clear and articulate sentence, formatting questions without hesitancy and learning how to phrase to get the best result* and the other respondent used it *as a way of helping them with their routines*, that is, *Alexa, tell me my schedule for today.* In this second case, the therapist noted how engagement with Alexa was seen to be particularly beneficial as “it is not a ‘disability’ device, it is something everybody is using.”

#### Dementia

Remaining within the space of cognitive impairment, there was one example of a therapist who had used Alexa with *patients with different types of dementia as a tool to organize diaries, timetabling, reminders, shopping lists, music, and Alexa to Alexa calls to family.* Although they reported “varying success due to personal preference, but also the trigger word Alexa can sometimes be difficult to remember,” they reported that the technology could be used successfully if individuals were supported, and the use of the device was modeled effectively. This therapist described “it can make a positive difference to them, maintaining independence, keeping in contact with people and for quality of life.”

#### English as a Second Language

Finally, 1 therapist described using Google translator to support a case history taking exercise with a family with limited English (as the translator had failed to arrive in time), so they had *the option of understanding in their home language.* They said, “I was able to complete a case history and save time by not having to re-book the assessment. The parents were very happy.” Although this example was not directly related to patient outcomes, it was a broader example of how this type of technology might be able to support the therapists themselves in their professional activities.

#### Reported Limitations

It is worth noting that although the reported use cases were vastly positive across the therapists, there were some instances where therapists discussed limitations with the technology (n=3). One therapist discussed how a client with Parkinson disease had “improved speed of task. However, fatigue impacted.” Another, discussing a wheelchair user with traumatic brain injury, had variable success: “useful but needs clear speech which is sometimes not clear enough.” Finally, 1 therapist discussed how 1 aphasic client they had been using Alexa with had “found it slightly useful but the quality of his wifi connection was poor and this impacted how well it worked for him.”

### Perceived Potential Benefits of VAT for the Wider SLT Community

We asked all 230 respondents, regardless of whether they had reported experiences of using VATs in their practice, whether they felt there were any possible (perceived) benefits of using VAT with their SLT clients. Although many participants remained unsure (potentially because of a lack of experience and training), the majority either agreed or strongly agreed that VAT could have a positive impact on their clients’ speech and confidence (Table 5).

**Table 5:** Respondents’ views on the potential impact of voice-assisted technology for their clients (N=230).

Statement	0 (strongly disagree)	1 (disagree)	2 (maybe, but I’m not sure)	3 (agree)	4 (strongly agree)
These technologies could have some impact on patients’ speech, n (%)	2 (0.9)	12 (5.2)	90 (39.1)	99 (43)	27 (11.7)
These technologies could help patients speak louder, n (%)	0 (0)	11 (4.8)	99 (43)	92 (40)	28 (12.2)
These technologies could help patients speak more clearly, n (%)	1 (0.4)	23 (10)	97 (42.2)	88 (38.3)	21 (9.1)
These technologies could increase patients’ confidence in their speech, n (%)	0 (0)	9 (3.9)	86 (37.4)	112 (48.7)	23 (10)

The respondents were asked to describe any other potential benefits that VAT could have on their clients; 175 (76.1%) of the 230 respondents provided additional information via free-text responses. Similar to the therapists who had experience using the technology, accessibility and independence, psychological benefits, and speech improvement were the major themes identified.

Many respondents (51/175, 29.1%) believed that VATs would enhance accessibility for their clients and give them more control over their environment. They provided examples, such as “It could help clients access online services, environmental controls and communication platforms to communicate face to face with others *e.g.* skype/facetime” and “It would also be beneficial if they were to have physical conditions which restrict their ability to stand to turn the TV/radio on.” Many respondents (35/175, 20%) explicitly stated that the technology would make their clients more independent. For example, 1 respondent stated, “It can help someone to be more independent and not rely on another person to meet their requests.” Improving their clients’ organizational skills, by setting up reminders or diaries easily, was also identified as a benefit by some respondents (16/175, 9.1%). They provided examples, such as “I think they could be used for prompts to remember to do things. Easier for people to be able to access information” and “Most of my patients have cognitive impairments as well as communication impairments - these technologies have the potential to be very helpful to someone with poor memory and orientation.”

Several respondents focused on the psychological benefits that VATs could have. Some respondents (19/175, 10.8%) stated that the devices provided a sense of normality and were not therapy devices, as they were something that everyone used. This reduced stigma and encouraged the clients to use the devices: “As the technology is mainstream, as is technology generally, it is more socially acceptable and less different and isolating for AAC users now than it has been in the past.” A few respondents (18/175, 10.3%) also mentioned the enjoyment and motivation effect of these devices, stating “Enjoyment and expansion of communication for clients that are non-verbal. It provides a sense of freedom outside of the structured AAC device.” Some respondents (11/175, 6.3%) also believed that the devices could enhance their clients’ communication and community engagement. They provided examples, such as *improved ability to communicate with others* and *communication at home/between family members/engaging more with the younger generation.*

Many respondents stated that the devices can have a direct impact on the speech of their clients. A few respondents (18/175, 10.3%) stated that using the devices could make speech more intelligible and clearer, by providing examples, such as “Encourages increased volume/clarity of speech” and “Enables users with degenerative conditions to maintain their voice for as long as possible.” Other respondents (19/175, 10.8%) mentioned how these devices could provide feedback on their clients’ speech and increase their self-awareness:

Impartial feedback from a non-human. If they haven't used speech sounds correctly then it's not a family member telling them. Enhances their own awareness of their intelligibility.

Awareness of the need to speak more clearly to be understood, feedback on intelligibility.

Finally, several others (15/175, 8.6%) stated how the devices could be used for home practice by their clients and provided examples, such as “Good for those who are socially isolated to practice speech” and “More inclined to practise at home where nobody else can hear them.”

## Discussion

### Principal Findings

#### Overview

The aim of this survey is to understand the attitudes and experiences of SaLTs toward VAT use. Specifically, we wanted to (1) develop an understanding of their use experiences (if any) and any potential benefits they might see in using the technology in the future and (2) understand their reasons for not using the technology and uncover any potential barriers that could be addressed in the future (eg, training needs). There has been no previous study on how VAT was used in SLT clinical practice, or indeed clinical practice, among other health professions. As such, the aim of this work is to gather a preliminary scoping overview of how SaLTs and their clients are currently using VAT. Our study findings will shape the future areas of research and identify potential clinical use cases that can be further developed.

Overall, 96.9% (223/230) of the respondents were women, which is unsurprising given that SLT is largely a female-dominated profession [38,39]. The respondents mostly worked within the UK NHS, with an even balance of early career and experienced practitioners. They demonstrated caseloads with service users possessing a diverse range of communication and swallowing needs. This indicates that the survey results expressed the views and experiences of a representative sample of SLT intervention areas. The respondents were very familiar with commercial VATs, with over 98.3% (226/230) having heard of Alexa and Siri. We found that over 21.3% (49/230) of our sample had already used VATs with a variety of different client groups and had a range of experiences to share.

#### Opportunities for VAT in SLT Practice

The therapists had already used VATs across 10 different client groups and provided detailed accounts of their use experiences and impacts. Almost half of these use cases involved using VAT to assist with day-to-day tasks, such as setting reminders, playing music, or sending electronic communications (emails and text messages) or controlling aspects of the clients’ environment. Several previous studies have discussed the accessibility benefits of VAT for different populations (such as older adults and people with sensory or physical disabilities) by assisting them with these types of tasks [26-31]. This previous research and our own findings in this sense are not unexpected, given that these are the very functions that VATs are marketed to perform, and are the intended commercial purposes of these devices. Although the accessibility benefits of these functions are undoubtedly useful to individuals requiring SLT, many of whom also have underlying physical disabilities, some of the respondents in our study described further innovative uses for VATs that were of particular interest.

Some of the respondents mentioned explicitly using the devices to target the practice of SLT strategies, with several client groups experiencing diagnoses, such as dysarthria, apraxia, and aphasia. These types of conditions are communication impairments that cause difficulties in producing clear, comprehensible speech. In the first instance, one might question how clients with speech impairments can successfully communicate with VAT, which requires specific trigger words and clear speech to function. However, the respondents explicitly discussed using VATs to support speech practice, particularly as a way to provide biofeedback to the client on their speech clarity. They highlighted the positive impacts of having a device that would provide such a source of feedback on speech production; if the device could understand a client, they were given clarification that they were speaking intelligibly. This echoes the findings of other studies, which have highlighted the successful experiences of users with speech impairment when using commercial VAT devices [27,28,32]. As highlighted by our professional SLT participants, the ability to practice speech at home and obtain real-time, impartial feedback from the devices was found to improve word production accuracy and increase clients' motivation to perform home therapy practice, both of which are SLT outcomes that might regularly be targeted in formal therapy. A study [27] also reported similar findings, discussing that some Alexa users self-reported speech improvements, with a need for distinct pronunciation when using the device actively improving users’ speech through continuous practice. Duffy et al [32] also found self-reported speech improvements in approximately 25% of people with Parkinson disease who were using VATs but did not delve into why participants felt this might be happening. This is an exciting and ripe area for future research, which might explore the extent to which speech changes actually occur through device use, how they are maintained, and how speech outcomes being achieved through device use might be measured in SLT practice.

Another significant use case discussed was of AAC users, wherein respondents described how they used a combination of digital AAC devices and VAT with their clients. They discussed how the computerized voice output of the digital AACs was used to access the functionalities of VAT, such as performing day-to-day tasks and environmental controls. They reported very positive impacts on the confidence, accessibility, and independence of their clients and highlighted how the social acceptability and mainstream nature of VATs helped reduce barriers to their use and increased the clients’ motivation to use them. As VAT is widely used in society today, therapists can present it as something that everyone is using, not an assistive or *disability device*. This social acceptance surrounding VATs has an inclusive effect and is less isolating for the client, in turn, having a motivating effect. Similar conclusions were drawn in another study [40], which discussed that the popularity of VAT devices added to the feelings of inclusion for people with cognitive and linguistic difficulties. It was also interesting to note how nonverbal users were being supported by therapists to use VAT by combining them with their AAC devices. Although there are a few practice-focused articles in the grey literature [41,42] that discuss this use case and its impact on user independence and motivation, formal research in this space is limited. Future research can further explore the experiences, benefits, and limitations of this integration with AAC users.

Considering the broader perspectives of our sample, 72.1% (166/230) of respondents who had not used VAT in practice were very keen on trying it in the future. This highlights the largely positive outlook and perception of the technology by SaLTs. They perceive multiple benefits of using VAT with their clients, such as increased accessibility, independence, and confidence. Moreover, these respondents also believed that the technology could have an impact on their clients’ speech. In total, 52.2% (120/230) of the respondents believed that the technology could help their clients speak louder, although 47.4% (109/230) of the respondents believed that it could help them speak more clearly. Most significantly, 58.7% (135/230) of the respondents stated that the technology could increase their clients’ confidence in their speech. These perceptions about the benefits resonate with our findings from the practitioners’ experiences. Respondents who had used VAT with their clients reported very similar benefits, as discussed earlier. Furthermore, these types of benefits have also been discussed in previous literature [27,28,32]. Our findings, backed by previous studies, demonstrate the potential of VAT to support SLT. Future research can explore how the technology can be leveraged to augment traditional SLT by providing users with an opportunity for home practice. However, there is work to be done around how different types of client groups might be best supported in their use as well as the types of activities that might be most beneficial to be conducted with VATs to benefit the service user.

Traditionally, SLT is delivered on an individual basis, face to face usually in clinical settings, but successful outcomes can depend on practice at home [43]. Home practice supports the carryover of skills from clinic to everyday life and contributes to the maintenance of communication improvement. Technology has been found to have the potential to address some of these issues and promote better self-management practices [16,25]. This can be particularly beneficial for clients living alone or in rural areas, where traveling to therapy appointments might be challenging and is perhaps even more important in the current context of the COVID-19 pandemic and lockdowns. VATs have the potential to improve users’ self-management practices by supporting the delivery of home-based therapy programs, with the benefit of immediate feedback from the device [44]. Designing such programs that can be facilitated by the qualities of VAT devices is an interesting area for future research. However, we do not suggest that this is a magical solution. Work in this space also needs to consider the role of the technology alongside timely therapist input so that effective long-term and multifaceted support can be provided to the client within such a technology-assisted service delivery model. In addition, researchers and clinicians should be aware of conditions that will see a decline in speech over time or in conditions such as Parkinson disease, where fluctuations might even be seen within the span of days or hours. These types of clients may find VAT interaction challenging and unpredictable and will need support from professionals to ensure their accessibility and reduce frustration.

#### Barriers to VAT Use in SLT Practice

Although the outlook toward VAT use was largely positive and several potential benefits were identified, the respondents also identified several barriers to adoption and concerns regarding its use. Many respondents described organizational and infrastructure barriers that could affect technology adoption, for example, the cost of devices; supporting infrastructure, such as internet connectivity; limited access to technology; and funding in their organization. Moreover, data collection and protection policies, such as the GDPR, were a concern for some of the participants. Respondents felt that the commercial VATs had unclear data collection and use policies, which would make them difficult to use in client-related contexts. These concerns are not new when it comes to discussing novel technologies and their integration into health care contexts. There has been a wealth of literature exploring the challenges of technology adoption and uptake by health care professionals [45-47]. Respondents discussed concerns about data storage, confidentiality, and privacy, which are always crucial considerations when using technology in health care contexts [32,48]. These are significant concerns to be considered in future work, which would need to explore how commercial VATs (and any new apps or skills to be developed to support clinical care) can be introduced and used in line with user privacy and confidentiality requirements. That said, work is beginning to emerge that is actively exploring the users’ perceptions of data and data sharing in relation to commercial devices used for health needs [29]. Transparency, openness, and information about what data are being stored and used and by whom, is enough to alleviate many people’s concerns (even if these data are being stored by a large-scale commercial company).

The respondents in our survey who had not used VAT reported multiple reasons for their lack of use. Most respondents (131/181, 72.4%) mentioned a lack of opportunity for use; however, lack of technology awareness and training were other significant reasons that were reported. Most respondents desired basic training about the range of technologies available, their use, potential benefits, and information about applying them in practice. Providing specific application examples in future training may be beneficial, as some respondents wanted information specific to a particular client group, citing examples such as dysarthria, AAC users, and dysfluency. As with any new technology, training before use is essential. Several health care studies have documented this concern [49,50]. Developing structured, co-designed training materials for VAT is essential for practitioner adoption and use. Delivering organization wide technology training may be an optimal solution for this issue. Previous literature has documented the success of delivering technology training with professionals within the space of telehealth [51,52] and virtual reality [53-55]. Future work could focus on developing formal VAT training resources and identifying the best methodologies to deliver this training effectively.

The technological limitations of VAT were another barrier identified by this study. Many respondents discussed how there were existing challenges related to VAT devices’ ability to even comprehend attempts produced by users without speech impairments. Failure to detect different accents, unclear speech, low-volume speech, and misinterpreting words were some of the issues respondents felt the technology was *inconsistent* and *temperamental* within its functional ability. In light of this issue, respondents felt that their clients with severe speech impairment would be unable to use VAT effectively, leading to negative impacts on confidence and motivation. There is a risk that such users would feel frustrated because of this issue, demotivating them from using such technology in the future. However, we found that respondents who had actually used the technology found high success rates with a variety of client groups, even clients with severe speech impairments. The respondents found workarounds to the technical limitations of the technology and stated how they managed to use VAT and foster motivation for their clients. This disparity between perceived barriers and actual experiences calls for additional research in this domain. It is possible that developing a community where practitioners can exchange their thoughts and experiences may be beneficial for wider VAT adoption. There are also opportunities for conducting research that would help with anticipating problems as well as developing bespoke skills, integrated apps, or new features for VATs that may provide solutions. Our findings suggest that different user groups will have different expectations, capabilities, and impacts of using VAT. As such, there is a direction for future research to focus on exploring how individual clients or client groups with different levels of speech impairment are able or unable to use VAT.

### Limitations

The study presented in this paper had several limitations that should be discussed. First, it was conducted exclusively with users based in the United Kingdom. Technological preferences, experiences, and outlooks differ across regions and countries, and the survey does not represent the overall global experiences of SaLTs. Future studies are required to understand the generalizability of our results.

Second, the survey was web-based and self-selective, implying that anyone could potentially provide a response. Naturally, the respondents had basic digital skills and were able to successfully navigate and answer the survey. As such, they may be biased toward the use of such technology and have a positive outlook toward its use. Other SaLTs who might not be technologically adept could have different perspectives and a less favorable outlook toward technology use. In addition, we did not explicitly ask the participants if their experiences were with current users of VAT or if they had introduced the technology to their clients. This information would have provided an additional context about use experiences. Clients already using the technology could have developed certain skills and have different perceptions compared with clients that were introduced to the technology for the first time.

Finally, the sample size estimated to obtain 90% confidence with –5% to +5% margin of error was 289. The number of participants who completed our survey was 230, which was somewhat lower than the estimated value. Time and resource constraints meant that we were unable to keep the survey open for longer. Offering respondents a financial benefit to participate in the study might have improved the speed of our uptake; however, this was not within the scope of our resources.

### Future Work

Our study highlights several clear directions for future research, which have been described in the discussion section. Our perspective is that the primary directions for future research should first focus on developing a focused understanding of VAT use within specific use case scenarios and understanding the best ways to collect and report upon potential clinical benefits that might be seen in these use cases. Second, work is required to develop VAT education and training to increase future uptake and adoption. Further work must be done to identify the optimal route to deliver this education and training to raise awareness of the potential benefits and confidence in use.

### Conclusions

VAT has been used by a number of UK-based SaLTs in clinical practice. Wider adoption of the technology is limited by the lack of professional opportunities, training, and understanding. Although other studies have explored the interaction between technology and several client groups, our study presents opportunities and challenges from the perspective of the practitioners. The data show increased engagement, empowerment, and the possibility of achieving therapeutic outcomes in clients with communication impairment. The disparate responses suggest that this area is ripe for the development of research exploring the role of VATs in evidence-based clinical practice, starting with a clear definition of its use potentials and benefits and the development of plans for outcome measurement when using VAT devices to support therapy aims.

## Abbreviations

AAC: augmentative and alternative communication
GDPR: General Data Protection Regulation
NHS: National Health Service
RCSLT: Royal College of Speech and Language Therapists
SaLT: speech and language therapist
SLT: speech and language therapy
VAT: voice-assisted technology
